# LAWTON’S THEORY OF PERSON-ENVIRONMENT FIT: THEORETICAL FOUNDATIONS FOR DETECTING TIPPING POINTS

**DOI:** 10.1093/geroni/igz038.2218

**Published:** 2019-11-08

**Authors:** Janice D Crist, Cheryl Lacasse, Linda R Phillips, Jian Liu

**Affiliations:** 1 University of Arizona, Tucson, Arizona, United States; 2 College of Nursing, The University of Arizona, Tucson, Arizona, United States; 3 Arizona Center on Aging, Tucson, Arizona, United States; 4 Department of Systems and Industrial Engineering , The University of Arizona, Tucson, Arizona, United States

## Abstract

Caregiving families often experience “tipping points,” changes that forever alter their lives, such as a fall with a fractured femur. Tipping points for older adults can be conceptualized as an interaction between individuals and their environments. According to Lawton’s theory of person-environment fit (Lawton, 1983, 1985), physical and social environments and the person’s behavior are shaped by one another in a dynamic, ever-changing process. For older adults, the relationship between “environmental press,” or the mismatch between the person and his/her environment, and adaptation to that environment is mediated through one’s ability to cope. When stressors in health, cognition, or caregiver availability occur, environmental press may heighten, leading to a tipping point. In this paper the authors clarify how environmental press theory provides a foundation for studying early detection of impending tipping points and facilitating decisional support of families for choosing the right long-term support services at the right time.

